# A Mixed-Methods Formative Evaluation of a Dementia-Friendly Congregation Program for Black Churches

**DOI:** 10.3390/ijerph19084498

**Published:** 2022-04-08

**Authors:** Janelle Gore, Jamilla Toliver, Miranda A. Moore, Dawn Aycock, Fayron Epps

**Affiliations:** 1 Nell Hodgson Woodruff School of Nursing, Emory University, Atlanta, GA 30322, USA; jamilla_carter@yahoo.com (J.T.); fepps@emory.edu (F.E.); 2 School of Medicine, Emory University, Atlanta, GA 30322, USA; miranda.moore@emory.edu; 3 Byrdine F. Lewis College of Nursing and Health Professions, Georgia State University, Atlanta, GA 30303, USA; daycock@gsu.edu

**Keywords:** African American, dementia, formative program evaluation, Black church

## Abstract

Black churches have traditionally been a haven for Black American families; however, many churches do not currently have programs to support families living with dementia. Alter™ was established to assist faith communities in meeting the needs of these families and becoming a viable resource to promote their health and wellness. Alter™ achieves this aim through a three-pronged approach: (1) conducting educational sessions, (2) modifying Black churches to be dementia-inclusive spaces, and (3) providing ongoing support. The principal goal of Alter™ is to offer guidance to churches in adapting their community to reflect a supportive environment for families affected by dementia. Alter™ uses a partnership checklist to encourage activities that incorporate dementia education opportunities, environmental modifications, and dementia resources and support. This paper reports on a formative mixed-methods evaluation of church partners enrolled in Alter™. Church partner ambassadors within the faith communities participated in the evaluation survey (*n* = 8) and two focus groups (*n* = 11). Ambassadors are appointed by church leadership to lead the implementation of program activities. Data were collected concerning the levels of helpfulness, difficulty, usefulness, or utility of checklist activities and modifications and the ease of program implementation. The evaluation also assessed the COVID-19 pandemic’s impact on each church’s ability to implement activities and modifications. The survey results revealed that most required partnership activities and modifications were found to be at least moderately helpful. Some of the items (themes) that differed across church partners included barriers to implementing activities, the support provided and needed, and the use of program funding. This evaluation provides key insights to consider in developing and refining community-based, dementia-friendly communities (including faith communities). As implementation science expands and improves, the need to evaluate the implementation of programs continues to be highlighted. Our formative evaluation shed light on key areas in which modifications to our original programming would lead to program improvement and sustainability. Additionally, implementing the modifications identified in our evaluation will facilitate the achievement of the mission of Alter™ to improve the well-being of older adults affected by dementia and their families. Other programs would reap substantial benefits from engaging in similar formative evaluation efforts.

## 1. Introduction

In 2021, experts estimated over 6 million Americans were living with Alzheimer’s disease and other dementias [1]. Among Americans affected by dementia, the rate of diagnosis, and prevalence of dementia among Black Americans are disproportionately increasing, compared with White Americans [1,2]. Unfortunately, Black American families affected by dementia are often hesitant to share the person living with dementia’s (PLWD) diagnosis with their community and seek supportive services due to the disease’s stigmatizing nature [3,4]. Black churches can play significant roles in establishing community norms, reducing dementia-related stigma, and increasing the well-being of families by attending to families’ spiritual, emotional, and social support needs [5,6,7,8,9]. In fact, Black churches serve as conduits for health promotion and programming in the African American community [6,9,10,11].

Churches have the capabilities to promote health within the community, communicate and disseminate health information, foster and maintain trusting, supportive environments for health, and offer relevant safe, culturally appropriate health prevention programs and care for the community without racial discrimination [6,9]. Furthermore, churches have a strong volunteer base that can assist in health promotion activities [12]. Moreover, church clergy can offer health resources that members may not have had access to and can play an essential role in influencing opinions and deciding which health resources and other programs are needed to bring to their respective churches [13].

Building dementia-friendly (i.e., supportive and inclusive) communities is a growing priority to meet the educational and infrastructural needs of families affected by dementia [14]. However, many Black churches do not currently have programs, infrastructure, or the knowledge of dementia resources to support and accommodate families affected by dementia [15]. Therefore, community programs such as Alter™ [16] guide Black churches in modifying their community to support and provide resources for families affected by dementia by encouraging churches to implement dementia-friendly initiatives. 

Currently, there is a growing literature surrounding the science of implementation and sustainability strategies of health promotion programs [17,18]. As healthcare professionals expand beyond implementing programs in clinical settings to community settings, it becomes increasingly important to evaluate a program’s individual outcomes (Eccles and Mittman, 2006). Therefore, it is essential for community organizations to understand the strategies and approaches to effectively implementing evidence-informed health programs [17]. To the authors’ knowledge, no formative evaluation has been conducted to assess the experiences of Black faith communities implementing an evidence-informed, dementia-friendly program at their church. Thus, this article will describe a formative program evaluation conducted to determine programmatic gaps with the Alter™ program related to supporting Black churches in becoming dementia-friendly communities.

## 2. Materials and Methods

### 2.1. Program Description

Alter™ is a faith-based, dementia-friendly community program that equips Black churches with the tools needed to better support families affected by dementia [16]. Alter™ achieves this aim through a three-pronged approach: (1) conducting educational sessions, (2) modifying Black churches to be dementia-inclusive spaces, and (3) providing ongoing support. The program recognizes the church as a member of the dementia care team and incorporates faith-based interventions to support dementia-affected families, both virtually and in person. Alter™ equips participating churches with dementia education resources, connects aging and dementia agencies to the church, assists with environmental modifications, and assists with the implementation or expansion of dementia support programs at the church.

### 2.2. Program Recruitment and Implementation

Churches were recruited utilizing snowball and purposive methods. Purposive methods were used to focus enrollment efforts on under-resourced communities. During the enrollment process of the program, respective church representatives met with the program staff for an informational meeting. Upon enrollment to become a partner, churches received a welcome box accompanied by a memorandum of understanding and a financial contribution to assist with the implementation of partnership initiatives. The Alter™ partnership initiatives checklist was inclusive of dementia-related initiatives and environmental modifications, which provided church partners the framework to offer dementia support resources, dementia education, and meaningful worship experiences. The partnership checklist was divided into four domains: core (*n* = 8), support (*n* = 6), community education and awareness (*n* = 4), and the worship experience (*n* = 6). Church partners agreed to implement at least 16 of the 24 suggested activities and modifications on the Alter™ partnership initiatives checklist over a 2-year period. The selection of activities to implement was performed by the respective church representative based on their ministry’s mission and strategic plan. The full list of activities included on the Alter™ partnership initiatives checklist is provided in Appendix A.

### 2.3. Evaluation Design and Measure

The evaluation team conducted a formative evaluation to understand church partners’ experiences implementing partnership checklist activities and modifications. Formative evaluation methods were utilized to assess a program’s goals, barriers and facilitators to implementation, and opportunities for program sustainability during the initial phases of program implementation [19,20]. This method was appropriate for the present evaluation due to Alter™ being implemented for no longer than a year and the collection of data being used to explore the current gaps within the implementation of the program. Evaluation questions and a mixed-methods approach were developed based on the program’s current implementation status and expressed needs from program stakeholders. Two main evaluation questions and three sub-questions were crafted specifically to understand the experiences of church partners implementing activities and modifications.

1. How can Alter™ best support partnering churches as the program continues to enroll more churches?
  - How helpful are the proposed program activities and modifications?
  - How difficult is it to implement the proposed program activities and modifications at partnering churches?
  - How useful are the proposed program activities and modifications?
2. How did the COVID-19 pandemic impact participating churches’ ability to participate in the program fully?

The evaluation survey (Supplementary Materials) was modeled after the partnership initiative checklist (Appendix A); therefore, the survey questions were grouped by the four main domains from the checklist (core, support, education and awareness, and the worship experience). An evaluation team member administered the surveys via a video-conferencing platform (Zoom) to collect church partners’ perceptions of activities based on their level of helpfulness, utility, and difficulty. The survey further assessed why selected activities were not yet implemented. Each church partner was asked to respond to 53 multiple-choice items and 4 open-ended questions to capture nuances with church partners’ experiences implementing the program at their respective churches. 

Focus groups were conducted via a video-conferencing platform to explore the findings of the surveys conducted with church partners and further assess support received from the Alter™ staff. The focus groups consisted of church partner representatives and were organized using the RE-AIM framework [21,22] (Appendix B). Using the framework, the implementation of Alter™ at participating churches and the impacts of COVID-19 were explored.

### 2.4. Data Collection and Procedures

A data collection crosswalk tool was created to map out domains that were assessed in data collection instruments (focus groups, survey) onto their corresponding evaluation questions. This process ensured that all data collected were valuable to answer the evaluation questions and addressed the overall purpose of the evaluation (Table 1). The principal investigator (PI), evaluator, program coordinator, and faculty member collaborated to write the survey based on the evaluation questions. The survey consisted of 106 questions, both open-ended and multiple-choice.

Program staff initially contacted church partners to inform them about the evaluation being conducted. The evaluation team contacted church partners with provided contact information to coordinate schedules and explain evaluation ethics, including that the survey and focus groups were voluntary and that responses were anonymous and would be kept confidential, and answered any questions from participants. The evaluation team then provided appropriate Zoom links for the survey and focus groups. The evaluator administered the survey, and their screen was shared with the participant using Zoom functionalities. All survey data were stored and secured on Qualtrics. An online survey was administered instead of a paper survey due to social distancing recommendations, thus eliminating the need for data entry. In addition, due to the guided nature of the survey, participants only needed to have an electronic device with Wi-Fi and Zoom capabilities. 

Focus group guides were developed based on preliminary results from the surveys in order to gain better insight into the overall experience of implementing Alter™ partnership initiative activities. All church points of contact participating in the evaluation survey were asked to schedule a time to participate in one of two focus groups. The program coordinator and program founder were also in attendance for both of the focus groups and helped guide the discussion. The focus groups occurred via Zoom and were led by the evaluation focus group guide and probes. Focus group sessions were transcribed verbatim, and all identifying information was removed.

### 2.5. Statistical Analysis 

Data from the church partner surveys were exported from Qualtrics into an Excel spreadsheet. Team members (J.G. and J. T.) cleaned all data within Excel. Closed-ended and open-ended data from the surveys were organized into separate Excel sheets for data analysis. The evaluation team ran descriptive statistics on the quantitative data to obtain frequencies and percentages for categorical data. A content analysis was completed on the open-ended survey responses to understand the impact of COVID-19 on the church’s abilities to conduct program activities and modifications. Once all the descriptive statistics and frequencies were reported, the data from the survey were triangulated to extract salient themes. 

### 2.6. Qualitative Analysis 

Transcribed focus group sessions were inductively and deductively coded to develop themes. First, an evaluation team member coded the transcripts based on previous knowledge. Then, the evaluator deductively coded, placing codes that align with the evaluation questions. The evaluator cross-analyzed the focus group data to formulate themes that were utilized during data triangulation. All codes and themes were presented to the other members of the program evaluation team and discussed for reliability. 

## 3. Results

### 3.1. Church Partner Characteristics

At the time of the program evaluation, nine faith communities were enrolled in the program (eight located in Georgia and one in Illinois). Of the nine church partners, eight completed the online survey. Church partners reported having majority Black congregations, and their congregant’s average socioeconomic status was middle income. Congregation sizes varied, as two reported having a small congregation (average weekly attendance of 50 or fewer people), five reported having a medium congregation (average weekly attendance of 51–300 people), and one reported having a “mega-church” size congregation (average weekly attendance of more than 2000). Overall, church partners described the age demographic of their congregation as the majority being older adults. 

### 3.2. Experience Implementing Program Activities and Modifications 

Data were reported based on participants’ experiences implementing selected program activities. Program activities consist of Core Partnership Activities (required for all partners to implement), providing support resources for families affected by dementia, providing community education opportunities to raise awareness about dementia, and implementing modifications to the worship service. Activities were assessed based on their level of helpfulness to the church becoming dementia-friendly, the difficulty of implementation, and the level of utilization (as appropriate). 

#### 3.2.1. Core Activities

Church partners were required to select and attempt to implement all core activities of assign to a minister, building is well-lit, with large print signage, badges for support staff, physical support, quiet room/space, accessible pathways, and church leaders workshop. As part of the core activities, all church partners assigned the dementia-friendly initiative to a ministry at their church. However, other core activities had a 73% implementation rate. Participants reported 62% of core activities as being “completely helpful” to their congregants and assisting with their church being dementia-friendly. The majority of churches (91%) reported core activities being “not difficult at all (easy)” to implement (Appendix C). Additionally, church partners (29%) reported “extensively utilizing” implemented core activities.

#### 3.2.2. Support Activities

Church partners were required to select and attempt at least three support activities. Four churches selected and implemented resource libraries and educational events, two churches offered support groups, one offered a respite care program, and one church offered social service coordination (Appendix D). Although five churches were selected to implement a Memory Café, none were able to implement the activity during this reporting period. Overall, 41% of the church partners reported being able to implement selected support activities at their respective churches. Church partners reported 33% of the selected support activities as being “completely helpful” to their congregation. The majority of churches (83%) reported support activities being “not difficult at all (easy)” to implement. However, church partners reported only “extensively utilizing” 1% of the support activities and modifications.

#### 3.2.3. Educational and Awareness Activities

Church partners were required to select and attempt at least two educational activities. Six churches selected and implemented the provision of dementia resources and education on social media/websites, four churches offered a Memory Sunday, and one church offered dementia education with youth (Appendix E). Although two churches selected a virtual dementia experience, none were able to implement the activity. Overall, 58% of church partners reported being able to implement selected education activities at their respective churches. Church partners reported 45% of selected education activities as being “completely helpful” to their congregations. The majority of churches (73%) reported education activities being “not difficult at all (easy)” to implement. Church partners reported “extensively utilizing” 27% of the educational activities and modifications.

#### 3.2.4. Worship Modifications 

Church partners were required to select and attempt at least three worship modification activities. All eight churches shortened their service length to 75 min or less, six churches simplified sermons, five increased the amount of music, five modified worship service order of flow, and three included familiar bible verses. One church selected to have a support staff present but was unable to implement this activity (Appendix F). Overall, worship activities had a 93% implementation rate. Church partners reported 59% of worship modifications as being “completely helpful” to their congregations. The majority of church partners (74%) reported worship activities as being “not difficult at all (easy)” to implement. Church partners (78%) reported “extensively utilizing” the worship activities and modifications.

#### 3.2.5. Barriers to Implementation

Church partners reported the COVID-19 pandemic as the main barrier to program implementation. Churches that were able to implement program activities reported conducting them before COVID-19 “stay at home orders”. Non-COVID-19 barriers were homogenous across most of the activities; therefore, we did not analyze barriers across activity types. The number of times a barrier was mentioned within the short answers of the survey was collected: the COVID-19 pandemic (f = 37); congregation members affected by dementia have not been identified (f = 7); lack of church personnel to conduct or lead activities (f = 7); unclear understanding of how to implement activities and modifications (f = 6); lack of marketing tools or knowledge of how to engage congregation members (f = 4); lack of space within the building to conduct activities (f = 3); perceiving the activity as not applicable for their church (f = 2); liability concerns (f = 1); not having enough funding to implement activities (f = 1).

### 3.3. Focus Group Findings 

All church partners participated in at least one of the focus group sessions. Church partners were able to have more than one representative in the focus groups; thus, 11 participants across all churches participated in the 2 focus groups. Three themes emerged during thematic analysis: barriers to implementing activities, the support provided and needed, and the use of program funding. The themes describe findings related to church partners’ experience participating in Alter™.

#### 3.3.1. Barriers to Implementing Activities

Participants expressed barriers to implementing activities while participating in Alter™. Barriers varied across churches; however, the COVID-19 pandemic presented as a significant barrier to implementing activities. Many church partners halted their implementation of various activities, as they were not able to be physically at the church. Participant 1 from focus group 2 expressed:

*“…once COVID came and then we shut everything down. So, we still have those things to do. You know? …, I think we would’ve been much further along had COVID not come.”*

Additionally, the COVID-19 pandemic presented other barriers, as it related to shifting the churches’ focus on prioritizing COIVD-19 information rather than disseminating information and resources related to dementia.

*“well, our organization have been so focused on trying to get back in the church as well as to bring education, ah, to their, ah, constituents, um, about COVID. And so, that’s been their primary focus and how they can, you know, really not lose their members during this time. … So, they have put the dementia back on the back burner, you know, during this time because that’s been their primary focus.”*

Participant 4, Focus Group 2

Churches also expressed other barriers related to the implementation of activities and modifications, including lack of space within the church building for activities, not knowing how to find or approach those affected by dementia within their church congregants, and perceived liability issues with implementing a respite program at the church. Additionally, church partners expressed the need for the program at their church but were not sure if their efforts were reaching families affected by dementia because many families are not forthcoming with sharing their diagnosis. However, church partners remained positive in their preparedness to support families affected by dementia when they step into their church.

*“But right now, we just really don’t have that many, ah, people in the congregation, ah, who are actually dealing with, or living with dementia … I’m sure people will be coming or, you know, or things will be changing. So, that’s where we are in getting more things set up so that when, um, they’re needed, they will be available.”*

Participant 2, Focus Group 1

Additionally, church participants noted there were activities they could have implemented, but they did not have a clear understanding of the activity. For instance, participant 2 from focus group 2 stated:

*“[W]hen we went through our survey [a] couple weeks ago, there were some things I think I could’ve implemented that I didn’t implement… I was like, “Oh, I… We could’ve did that.”*

#### 3.3.2. Support Provided and Needed

Participants detailed their experience receiving support from program staff and provided suggestions for improvements. Due to the COVID-19 pandemic, the support provided by Alter™ staff was virtual (e.g., Zoom, email, phone). Many church partners expressed their appreciation to the program staff for the attentive support provided:

*“Keep on doing the good job that you’re doing in helping us to become, ah, great Faith Village [dementia-friendly] churches. Because, ah, it’s just a, it’s something that’s very much needed and, ah, the team has done an excellent job even, ah, with the COVID situation.”*

Participant 1, Focus group 1

A participant recalled a time when an Alter™ staff member connected a family at a partnering church with dementia support resources.

*“Because the other day we had, um, someone and, and the, the father is member of our church. He’s, he’s older, but was doing very well, but has now moved back in with his daughter. And she had, um, she’s- she’s having to work from home and just some other things. But, ah, and I was able to, ah, connect her with [program staff], and it just really helped. Because we don’t know, well, let me speak for me. I don’t know all of the ins and outs and what may be available. I know with, with some of the information, but as far as talking to someone and seeing exactly where they are and what they need, then having her there has been just a tremendous asset.”*

Participant 2, Focus group 1

Participants discussed the possibility of Alter™ incorporating a direct phone line, through which churches can call and receive dementia-related information for their congregation members. Participants agreed to have a point of contact who understands the needs of their congregations rather than someone who does not know the church well is vital. For instance, Participant 2 from Focus group 1 stated:

*“Because we’re dealing with people who—in most cases—are older. [They wonder,] “Okay, I’ve gotta dial a 1–800 number. What…?” The first thing that comes to their mind [is], “Okay, I may get in a loop… You know, it’s gonna be somebody who doesn’t necessarily care about me or whatever.” … but having you contact the sister the other day and being able to talk to her, letting her know you understood exactly what she was looking for, and I don’t even know if she found everything, but it was the fact that this is somebody who understands what’s happening here.”*

During evaluation focus groups, church representatives were able to exchange ideas and discuss challenges in implementing various activities. Participants appreciated being able to gather and discuss what each church has done for the dementia-friendly activities.

*“But I think it, it’s, you know, it’s good that we are talking and getting- getting to, to know each other… and or- or call or whatever and say, “Well, how did… how did this work for you? This is what we’re trying to do, but we’re running into a roadblock.” You know? And, and sometimes just-just having that, ah, because it’s just, it’s a lot we can do but then it’s a lot that we can’t do.”*

Participant 2, Focus group 1

#### 3.3.3. Use of Program Funding

Alter™ provides a financial contribution to church partners to assist in the implementation of activities and modifications. Many church partners expressed challenges in spending the funding received. Although church partners have ideas about how to spend their funds, the COVID-19 pandemic presented a barrier due to many modifications not being able to be implemented.

*“Well, because of, ah, most of the churches been closed, ah, we haven’t used the money, ah, in The Alliance … So, um, it’s been beneficial, um, that, you know, we just hadn’t decided to use the money right now. We just doing, you know, we just doing what we do.”*

Participant 4, Focus Group 1

Furthermore, participants expressed not spending their funds due to not being in the building and only focusing on activities that can be conducted virtually.

*“…but in the future. the church is not that big. Obviously, um, we do not have anything that we are doing that’s costing anything right now.”*

Participant 1, Focus Group 2

Although many church partners expressed not being able to utilize program funds, one participant detailed their experience utilizing the funds to implement a caregiver support group and providing caregiver “support boxes” at their church.

*“And the toolbox had socks; it had, um, mints, nuts, um, a puzzle, a cross--no, word, word search puzzle. It had colored pencils for the colored, um, booklets. And just so happen, I went to the Dollar Tree, and they had those inspirational coloring books, and you could download the music, there was music to go along with the coloring books.”*

Participant 3, Focus Group 2

## 4. Discussion

Alter™ aims to provide a guiding light for faith communities in creating a dementia-friendly and inclusive environment. Informing Black churches of all sizes about the potential health needs of their communities, identifying church ambassadors and ministries who can implement health education initiatives, and providing churches with resources can be beneficial in building faith-based health programs [6,23]. Our findings are generalizable to any community-related health program, no matter the disease focus or the population served. Specifically, we found that actions related to implementation support, including additional assessments and resources, were needed for program adaptation and successful execution of activities. 

### 4.1. Adapting Health Promotion Programs Virtually

On average, more than half of the churches implemented the core, support, and education activities and worship modifications listed in the checklist. We anticipated higher implementation rates; however, barriers, particularly COVID-19, deterred or delayed activities that required being in the church building, such as a quiet room or space for persons with dementia to worship, memory cafés, and onsite education events, including conducting education with youth. The Reframing Aging Initiative, a collaboration of aging organizations, encourages aging professionals to “innovate existing systems to reduce poor health outcomes during and after the pandemic” [24]. Many Aging programs have answered the call by adapting existing programming to be disseminated via online platforms [25]. Virtual dissemination has allowed many health promotion programs to continue. Virtual dissemination has expanded programmatic reach geographically, broken economic barriers, and allowed for tailored programming [25]. However, establishing a plan to enhance online technical support is imperative as the health promotion landscape extends within a virtual world.

### 4.2. Assessing Community Organization Capacity for Programming

Health promotion programs seeking to address obstacles related to program sustainability would benefit from the development of an intake assessment tool measuring the need for their program activities and the capacity of their community partners to implement activities. Such a tool may ensure that community partners are able to implement selected activities to their intended extent. Assessing a community partner’s (Black church communities in the case of the Alter program) organizational readiness is essential for successful implementation [26,27]. An intake assessment may reveal which program activities are most appropriate for individual community partners to implement and allow program staff to appropriately allocate resources [28]. Additionally, by developing an intake tool, community partners may feel more confident executing certain activities based on their strengths, such as building infrastructure and organizational personnel. To this end, the assessment tool may also increase the implementation rates of activities selected while improving partner engagement. 

### 4.3. Developing a Program Manual

The level of helpfulness and usefulness of initiative activities perceived by church partners varied. Additionally, church partners expressed varying understanding of how to implement certain checklist activities and modifications. Thus, we believe providing community partners with a manual detailing how to implement program activities as well as expected outcomes may improve implementation efforts. For instance, a study assessing the impact of school-based education programs found successful interventions provided program implementors (i.e., church partners) with a program manual [29]. Additionally, a reference tool to help guide and advise community partners on the purchase of items needed to implement activities may be beneficial and address the focus group concern of “Use of Program Funding”. Furthermore, such a manual could serve as a recruitment tool. The manual would allow potential community partners to imagine how the program could be implemented within their organizations and populations.

### 4.4. Conducting Structured Workshops for Program Implementors 

Church partners found value in the focus groups, as they could hear from other church partners about their challenges and successes. Similarly, designating structured time for program site leaders to discuss their experience in a program has been utilized within pediatric health interventions to guide individual site improvements [30]. Thus, it may be beneficial to conduct three annual structured workshops focusing on self-assessment, strategies for improving dementia support, creating improvement plans, planning and implementing programs, utilizing resources, financial planning of stipends, and developing church policies to aid in sustainability [30]. Furthermore, workshops can provide a supportive network to enrolled community partners and boost the morale of those involved. 

### 4.5. Hiring Health Educators 

Ultimately, providing regular tailored support for community partners, and ensuring the organization is equipped with appropriate personnel such as health educators, is vital to a health promotion program’s success [31]. Health educators play significant roles in addressing public health issues. Health educators can answer questions, collect program data, and provide training and education, advice and referrals, and technical assistance to troubleshoot problems [30,31]. As programs grow, tailored and timely support can facilitate program sustainability. Dedicating funding to hiring support staff, and assigning them to specific partners as the designated point of contact may be an effective way to offer tailored support and increased utility of activities. 

### 4.6. Strengths and Limitations 

The mixed-methods approach was a strength of this evaluation, providing an overview and detailed information. Additionally, the majority (83%) of participating churches in Alter™ completed the evaluation activities. However, the evaluation had notable limitations. One limitation of the structured interviews was participant bias when responding to survey and focus group questions. Several church partners were familiar with the principal investigator (F.E.), which may have caused them to refrain from offering harsh criticism upon answering specific questions. Participant recall bias was another limitation; however, due to administering the survey and focus groups at a similar stage among all participants, recall bias may have been minimized [32]. Additionally, the COVID-19 pandemic added additional challenges to the program’s implementation and required the evaluation team to collect all data virtually. Furthermore, the evaluation experienced attrition of participants, as not all church partners participated in all aspects of the evaluation. 

## 5. Conclusions

To enhance, expand, and sustain community-based programs, program leaders need to engage in rigorous evaluation of their community-based partners’ perceptions of the program’s implementation barriers and facilitators. Our evaluation of the Alter program revealed strategies to overcome identified barriers—namely, adapting health promotion programs virtually, assessing community organization capacity for programming, developing a program manual, conducting structured workshops for program implementors, and hiring health educators. These strategies will help ensure effective implementation for any program seeking to partner with a community-based organization. 

## Figures and Tables

**Table 1:** Data collection cross-walk.

Evaluation Question	Domain Assessed
*Main:* How can Alter™ best support partnering churches as the program continues to enroll more churches?	Focus group data about support from staff and the overall percentages of the activities and modifications
*Sub:* How helpful are the proposed program activities and modifications?	Percentage of activities identified as helpful to church and congregation members
*Sub:* How difficult is it to implement the proposed program activities and modifications at partnering churches?	Percentage of activities identified as difficult to implement for the church
*Sub:* How useful are the proposed program activities and modifications?	Percentage of activities identified as being used and how often they were used
*Main:* How did the COVID-19 pandemic impact participating churches’ ability to participate in the program fully?	Content analysis of survey short answer responses and focus groups. Number of times COVID-19 is referenced as impacting program activities or modifications

**Figure A1:**
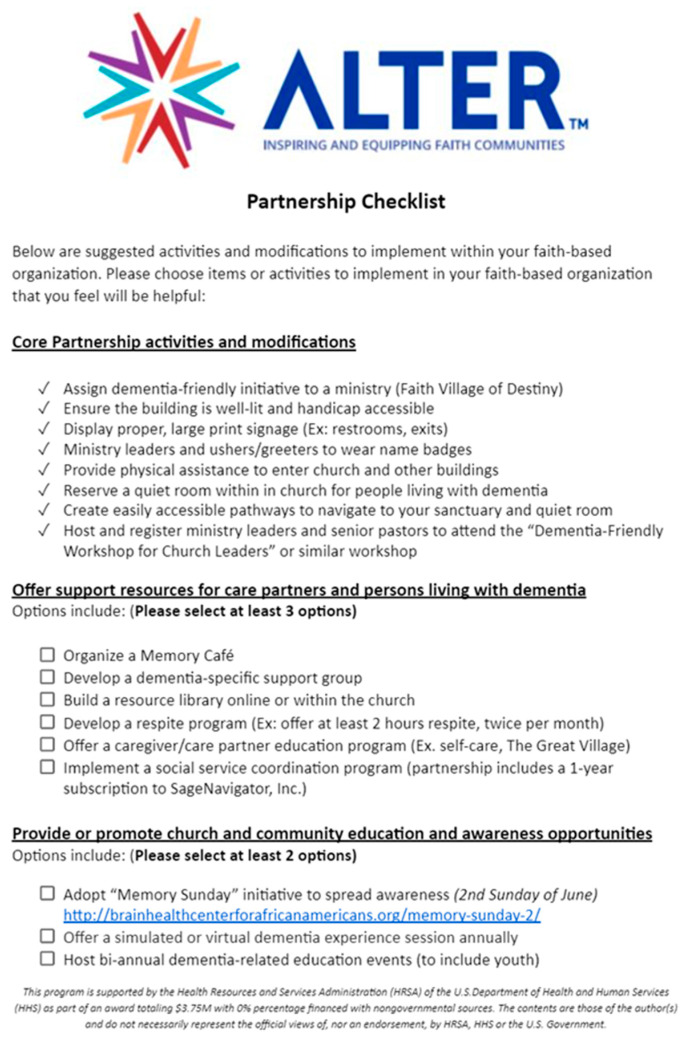

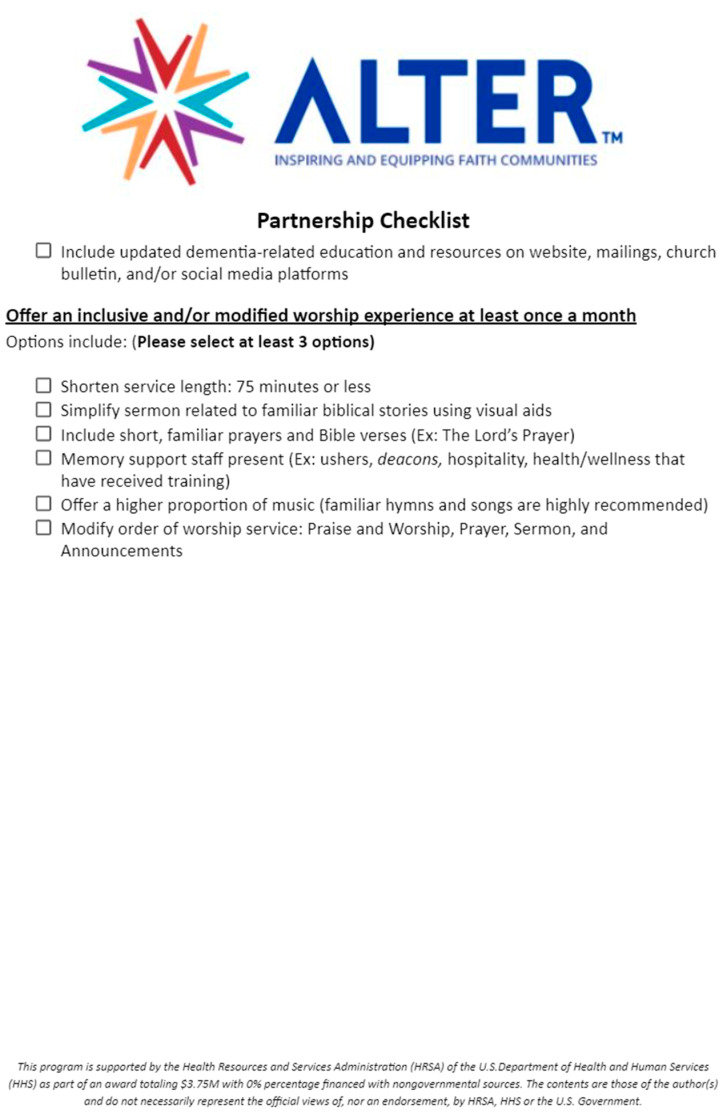
Alter Partnership Checklist.

## Data Availability

Not applicable.

## Supplementary Materials

The following supporting information can be downloaded at: https://www.mdpi.com/article/10.3390/ijerph19084498/s1, evaluation survey.

## Appendix B. Focus Group Guide (Utilized RE-AIM Framework)

**Reach:**

1. How did your church hear about the Dementia-Friendly Faith Villages Community program? Disclaimer: Dementia-friendly faith village community will be referred to as DFFVC throughout the remainder of the focus group.
2. Why did your church decide to participate in the program?
  - What were your churches first impressions of the DFFVC program?
  - Do you see this program helping other churches?
  - What attracted you to the program or the concept of dementia-friendly churches?
3. Can you all describe what the “Dementia-friendly faith villages community program” means to everyone’s respective church?

**Retention:**

4. Can you express to me how the program has helped/assisted your congregation and the church with creating a dementia-friendly church?
  - Do you feel the church is more educated?
  - How do you feel the church is more aware?
    - Explain why or/and example.
5. What elements of the program allow the church to continue to implement and modify the space into a dementia-friendly environment?
  - Structure, experience with staff, support
6. Is there something you would change about the dementia-friendly community program?
  - How would the changes you suggest impact the church members affected by dementia?
  - What would you like to see change within your church as a result of creating a dementia-friendly space?

**Effectiveness:**

7. Are there ways in which this program has had a positive impact on your church and church members?
  - Persons experiencing Dementia
  - Caregivers
  - Other congregants
8. Did implementing the DFFVC program present any challenges?
  - What aspects of the program are challenging?
  - Have there been challenges implementing the program? Why have you not implemented anything?
  - What other factors made it challenging? (i.e., Financial strain?)
  - What aspects have negatively impacted the church, caregivers, and/or persons living with dementia?

**Adoption**

9. How does the dementia-friendly program align with your churches’ mission?
  - Have you had to adjust your mission to fulfill the requirements of the dementia-friendly program?
  - Have you changed the dementia-friendly program to align with your churches mission?
  - How have you used the DFFVC program’s framework to make decisions for your churches when it comes to providing support and an appropriate worship experience for persons with dementia and their caregivers?

**Implementation:**

10. What have you seen as a result of implementing the DFFVC program?
  - Tell me about a time when you saw the program help someone or someone expressed gratitude about the program?
    - Any Success stories from anything you have implemented?
  - What is the program doing well?
11. We are always looking to expand the number of churches within our network. How do you think this program would benefit future churches?

**Maintenance:**

12. Do you feel the money received from the program to implement the dementia-friendly modifications assist your church effectively?
  - Explain why and how?
  - What are additional costs the church had to take on that the scholarship did not cover?
13. How do your churches plan to maintain the program in the next 2–3 years?
  - What is sustainable about the program?
  - What do you feel you will be able to maintain?
  - What factors may hinder the maintenance of the program?

**Conclusion**

14. What are additional ways we can support your church in creating a dementia-friendly space?

## Appendix C

**Table A1:** Core partnership activities.

Evaluation Survey Components	Assign to a Ministry	Building Is Well-Lit	Large Print Signage	Badges for Support Staff	Physical Support	Quiet Room/Space	Accessible Pathways	Church Leaders Workshop
**Implementation status**								
Not Selected	--	--	--	--	--	--	--	--
Selected to Implement and NOT Implemented		1	1	4	1	4	3	3
Selected to Implement and Implemented	8	7	7	4	7	4	5	5
**Level of helpfulness**								
Completely helpful	4	4	5	3	5	2	2	4
Very helpful	2	3	1	1	1	1	1	1
Moderately helpful	2	--	--	--	1	--	--	--
Slightly helpful	--	--	2	--	--	--	--	--
Not helpful at all	--	--	--	--	--	--	--	--
**Level of difficulty**								
Not Difficult at All	7	7	7	4	6	3	5	4
Slightly Difficult		--	--	--	1	--	--	--
Moderately Difficult	1	--	--	--	--	--	--	--
Very Difficult	--	--	--	--	--	--	--	--
Extremely Difficult (not able to implement)	--	--	--	--	--	--	--	--
**Utility**								
Extensively Utilizing	1	--	--	4	5	1	1	2
Utilizing	3	--	--	--	--	--	--	3
Moderately Utilizing	1	--	--	--	1	1	--	--
Somewhat Utilizing	3	--	--	--	--	--	1	--
Not Utilizing	--	--	--	--	1	1	3	--

## Appendix D

**Table A2:** Support resources for families affected by dementia frequencies table.

Evaluation Survey Components	Memory Café	Support Group	Resource Library	Respite Care	Educational Events	Social Service Coordination
**Implementation status**						
Not Selected to Implement	3	4	1	6	--	5
Selected to Implement and NOT Implemented	5	2	3	1	4	2
Selected to Implement and Implemented	--	2	4	1	4	1
**Level of helpfulness**						
Completely helpful	--	--	3	--	1	--
Very helpful	--	1	--	--	3	1
Moderately helpful	--		1	1	--	--
Slightly helpful	--		--	--	--	--
Not helpful at all	--		--	--	--	--
**Level of difficulty**						
Not Difficult at All	--	2	4	1	3	--
Slightly Difficult	--	--	--	--	--	1
Moderately Difficult	--	--	--	--	1	--
Very Difficult	--	--	--	--	--	--
Extremely Difficult (not able to implement)	--	--	--	--	--	--
**Utility**						
Extensively Utilizing	--	--	--	--	1	--
Utilizing	--	--	--	--	--	--
Moderately Utilizing	--	--	3	--	1	1
Somewhat Utilizing	--	1	1	--	2	--
Not Utilizing	--	1	--	1	--	--

## Appendix E

**Table A3:** Community dementia education and awareness activities frequencies table.

Evaluation Survey Components	Memory Sunday	Dementia Education with Youth	Dementia Resources and Education on Social Media/Website	Virtual Dementia Experience
**Implementation status**				
Not Selected to Implement	2	4	1	6
Selected to Implement and NOT Implemented	2	3	1	2
Selected to Implement and Implemented	4	1	6	--
**Level of helpfulness**				
Completely helpful	2	--	3	--
Very helpful	1	--	2	--
Moderately helpful	1	--	--	--
Slightly helpful	--	--	--	--
Not helpful at all	--	1	1	--
**Level of difficulty**				
Not Difficult at All	4	--	4	--
Slightly Difficult	--	--	1	--
Moderately Difficult	--	1	1	--
Very Difficult	--	--	--	--
Extremely Difficult (not able to implement)	--	--	--	--
**Utility**				
Extensively Utilizing	--	--	3	--
Utilizing	1	--	1	--
Moderately Utilizing	1	1	1	--
Somewhat Utilizing	--	--	--	--
Not Utilizing	1	--	1	--

## Appendix F

**Table A4:** Modified worship service activities frequencies table.

Evaluation Survey Components	Shorten Service Length to 75 min. or Less	Simplify Sermons	Include Familiar Bible Verses	Support Staff Present	Increase Amount of Music	Modify Worship Service Order of Flow
**Implementation status**						
Not Selected to Implement	--	1	5	7	3	3
Selected to Implement and NOT Implemented	--	1	--	1	--	--
Selected to Implement and Implemented	8	6	3	--	5	5
**Level of helpfulness**						
Completely helpful	4	3	2	--	3	4
Very helpful	1	2	1	--	2	1
Moderately helpful	1	1	--	--	--	--
Slightly helpful	--	--	--	--	--	--
Not helpful at all						
**Level of difficulty**						
Not Difficult at All	4	6	3	--	4	3
Slightly Difficult	2	--	--	--	1	1
Moderately Difficult	--	--	--	--	--	--
Very Difficult	--	--	--	--	--	--
Extremely Difficult (not able to implement)						
**Utility**						
Extensively Utilizing	6	4	3	--	3	5
Utilizing	--	--	--	--	--	--
Moderately Utilizing	--	1	--	--	2	--
Somewhat Utilizing	--	1	--	--	--	--
Not Utilizing	--	--	--	--	--	--
