# Supplementary material for: A Mixed-Methods Formative Evaluation of a Dementia-Friendly Congregation Program for Black Churches
Source: Int J Environ Res Public Health. 2022 Apr 8;19(8):4498. doi: 10.3390/ijerph19084498 (PMC9028238; doi:10.3390/ijerph19084498)
Supplement: Supplementary file 1 [file ijerph-19-04498-s001.zip › ijerph-1654159-supplementary.pdf]

ALTER

Introduction

The survey will be a "web-based" format and will take approximately 45-60 minutes for you and I to complete. If you choose to complete the survey, your responses will be kept confidential and any identifying information provided will be separated and stored separately from your survey responses. By completing this survey, you will be consenting for us to use your responses in this program evaluation.

Voluntary Participation and Withdrawal

Participation is voluntary. You may skip questions or stop participating at any time.

Contact Information

Contact Dr. Aycock (Site Principal Investigator) at 404-413-1178 & daycock@gsu.edu or Dr. Epps (Co-Investigator)

at 404-727-6936 & fepps@emory.edu

Consent

If you are willing to volunteer for this evaluation, then lets start the survey!

Thank you for participating in ALTER program evaluation. This program evaluation survey's goal is to gain an understanding of how the ALTER program is performing and how the program can better support your church and other churches within the network towards creating dementia-friendly spaces.

The survey is composed of 5 parts: *Required partnership activities and modifications, Offer support resources for care partners and persons living with dementia, Provide or promote church and community education and awareness opportunities, Offer an inclusive and/or modified worship experience at least once a month, Other customized activities*

To help ensure proper evaluation please answer each part and question of the survey to the best of your ability.

Thank you again for your time and participation!

Church information

Are you the point of contact from your church for ALTER?

- ○ Yes
- ○ No

Please list the name of the church you are completing the survey for?

[ ]

Date enrolled in the program: (Month/Day/Year) If you do not know the exact date approximate.

[ ]

Which choice best describes the churches size?

- ○ Mega church: Average weekend attendance more than 2,000 people
- ○ Large church: Average weekend attendance between 301 and 2,000 people
- ○ Medium church: Average weekend attendance between 51 and 300 people
- ○ Small church: Average weekend attendance 50 or fewer people

How would you describe the church community's demographic makeup? (church's majority age group, racial background or economic background)

Required partnership activities and modifications evaluation measures

The first part of the program evaluation is to evaluate the operation of the "Required partnership activities and modifications".

Has the church assigned the dementia-friendly initiative to a ministry?

- Yes
- No

Please select what summarizes the reason(s) why the church has not assigned the dementia-friendly initiative to a ministry? If there are any other or additional reason(s) please specify in the space below.

- ☐ Perceived as **difficult** to implement for the church in creating a dementia-friendly space
- ☐ Perceived as **not being useful** to the church in creating a dementia-friendly space
- ☐ Perceived as **not being helpful** to the church in creating a dementia-friendly space
- ☐ [ ] Other or additional reason(s)

Please list the name of the ministry the dementia-friendly initiative is assigned to:

[ ]

Since assigning the dementia-friendly initiative to a ministry, how helpful has that been for your faith-based organization (church and church members)?

- ○ Not at all Helpful
- ○ Slightly Helpful

- Moderately Helpful
- Very Helpful
- Completely Helpful

Since assigning the dementia-friendly initiative to a ministry, what was the level of difficulty for your faith-based organization (church and church members) to assign the dementia-friendly ministry?

- Extremely Difficult (not able to do so)
- Very Difficult
- Moderately Difficult
- Slightly Difficult
- Not Difficult at All (easy)

Since assigning the dementia-friendly initiative to a ministry, what best describes the level of uptake/usage for your faith-based organization (church and church members) with the dementia-friendly ministry?

- Not Utilizing the Ministry at All
- Somewhat Utilizing the Ministry (conduct meetings twice a year)
- Moderately Utilizing the Ministry (conduct meetings every other month)
- Utilizing the Ministry (conduct meetings every once a month)

○ Extensively utilizing the Ministry (have a prominence within the church consistently having meetings compared to other highly utilized ministries at the faith-based organization or church)

any additional feedback not captured in the above questions.

Has the church ensured your building is well-lit and handicap accessible?

○ Yes

○ No

Please select what summarizes the reason(s) why the church has not ensured your building is well-lit and handicap accessible? If there are any other or additional reason(s) please specify in the space below.

- ☐ Perceived as **difficult** to implement for the church in creating a dementia-friendly space
- ☐ Perceived as **not being useful** to the church in creating a dementia-friendly space
- ☐ Perceived as **not being helpful** to the church in creating a dementia-friendly space
- ☐ [ ] Other or additional reason(s)

Since ensuring your building is well-lit and handicap accessible, how helpful has that been for your faith-based organization (church and church members)?

- ○ Not at all Helpful
- ○ Slightly Helpful
- ○ Moderately Helpful
- ○ Very Helpful
- ○ Completely Helpful

Since ensuring your building is well-lit and handicap accessible, what was the level of difficulty for your faith-based organization (church and church members) to make and ensure these building adjustments?

- ○ Extremely Difficult (not able to do so)
- ○ Very Difficult

- ○ Moderately Difficult
- ○ Slightly Difficult
- ○ Not Difficult at All (easy)

any additional feedback not captured in the above questions.

Has the church displayed proper, large print signage (Ex: restrooms, exits, communications, in-building directions)?

- ○ Yes
- ○ No

Please select what summarizes the reason(s) why the church has not displayed proper, large print signage (Ex: restrooms, exits, communications, in-building directions)? If there are any other or additional reason(s) please specify in the space below.

- ☐ Perceived as **difficult** to implement for the church in creating a dementia-friendly space

- [ ] Perceived as **not being useful** to the church in creating a dementia-friendly space
- [ ] Perceived as **not being helpful** to the church in creating a dementia-friendly space
- [ ] ________ Other or additional reason(s)

Since displaying proper, large print signage (Ex: restrooms, exits, communications, in-building directions), how helpful has this been for your faith-based organization (church and church members)?

- ○ Not at all Helpful
- ○ Slightly Helpful
- ○ Moderately Helpful
- ○ Very Helpful
- ○ Completely Helpful

When displaying proper, large print signage (Ex: restrooms, exits, communications, in-building directions), what is the level of difficulty for your faith-based organization (church and church members) to make and ensure these signage adjustments?

- ○ Extremely Difficult (not able to do so)
- ○ Very Difficult
- ○ Moderately Difficult
- ○ Slightly Difficult
- ○ Not Difficult at All (easy)

any additional feedback not captured in the above questions.

Has the church required Ministry leaders and ushers/greeters to wear name badges?

- ○ Yes
- ○ No

Please select what summarizes the reason(s) why the church has not required Ministry leaders and ushers/greeters to wear name badges? If there are any other or additional reason(s) please specify in the space below.

- ☐ Perceived as **difficult** to implement for the church in creating a dementia-friendly space
- ☐ Perceived as **not being useful** to the church in creating a dementia-friendly space
- ☐ Perceived as **not being helpful** to the church in creating a dementia-friendly space
- ☐ ________ Other or additional reason(s)

Since requiring Ministry leaders and ushers/greeters to wear name badges, how helpful has this been for your faith-based organization (church and church members)?

- ○ Not at all Helpful
- ○ Slightly Helpful
- ○ Moderately Helpful
- ○ Very Helpful
- ○ Completely Helpful

When requiring Ministry leaders and ushers/greeters to wear name badges, what is the level of difficulty for your faith-based organization (church and church members) to make and ensure implementation of name badges?

- Extremely Difficult (not able to do so)
- Very Difficult
- Moderately Difficult
- Slightly Difficult
- Not Difficult at All (easy)

Since requiring Ministry leaders and ushers/greeters to wear name badges, what best describes the level of usage for your faith-based organization (church and church members) of wearing name badges?

- Not Utilizing Name Badges (users/greeters do not wear name badges)
- Somewhat Utilizing Name Badges (most users/greeters do not wear name badges during service)
- Moderately Utilizing Name Badges (about half the ushers/greeters wear name badges during service)
- Utilizing Name Badges (most ushers/greeters wear name badges during service)
- Extensively Utilizing Name Badges (all ushers/greeters always wear name badges during service)

any additional feedback not captured in the above questions.

Has the church provided physical assistance to enter church and other buildings?

- Yes
- No

Please select what summarizes the reason(s) why the church does not provide physical assistance to enter church and other buildings? If there are any other or additional reason(s) please specify in the space below.

- Perceived as **difficult** to implement for the church in creating a dementia-friendly space
- Perceived as **not being useful** to the church in creating a dementia-friendly space

- [ ] Perceived as **not being helpful** to the church in creating a dementia-friendly space
- [ ] [ ] Other or additional reason(s)

Since providing physical assistance to enter church and other buildings, how helpful has this been for your faith-based organization (church and church members)?

- ○ Not at all Helpful
- ○ Slightly Helpful
- ○ Moderately Helpful
- ○ Very Helpful
- ○ Completely Helpful

When providing physical assistance to enter church and other buildings, what is the level of difficulty for your faith-based organization (church and church members) to make and ensure physical assistance is provided?

- ○ Extremely Difficult (not able to do so)
- ○ Very Difficult
- ○ Moderately Difficult
- ○ Slightly Difficult
- ○ Not Difficult at All (easy)

Since providing physical assistance to enter church and other buildings, what best describes the level of uptake/usage of your faith-based organization (church and church members)?

- Not Utilizing (church members in need of physical assistance do not use the assistance)
- Somewhat Utilizing (church members in need of physical assistance mostly do not use the assistance)
- Moderately Utilizing (about half the church members in need of physical assistance use the assistance)
- Utilizing (most of the church members in need of physical assistance use the assistance)
- Extensively Utilizing (all the church members in need of physical assistance use the assistance)

any additional feedback not captured in the above questions.

Has the church reserved a quiet room within in the church for people living with dementia?

- ○ Yes
- ○ No

Please select what summarizes the reason(s) why the church has not reserved a quiet room within in the church for people living with dementia? If there are any other or additional reason(s) please specify in the space below.

- ☐ Perceived as **difficult** to implement for the church in creating a dementia-friendly space
- ☐ Perceived as **not being useful** to the church in creating a dementia-friendly space
- ☐ Perceived as **not being helpful** to the church in creating a dementia-friendly space
- ☐ [ ] Other or additional reason(s)

Since reserving a quiet room within in the church for people living with dementia, how helpful has this been for your faith-based organization (church and church members)?

- ○ Not at all Helpful
- ○ Slightly Helpful

- Moderately Helpful
- Very Helpful
- Completely Helpful

When reserving a quiet room within in the church for people living with dementia, what is the level of difficulty for your faith-based organization (church and church members) to ensure a quiet room is always reserved for those living with dementia?

- Extremely Difficult (not able to do so)
- Very Difficult
- Moderately Difficult
- Slightly Difficult
- Not Difficult at All (easy)

Since reserving a quiet room within in the church for people living with dementia, what best describes the level of uptake/usage of the quiet room from persons living with dementia at your faith-based organization (church and church members)?

- Not Utilizing the Quite Room
- Somewhat Utilizing the Quite Room
- Moderately Utilizing the Quite Room

- Utilizing the Quite Room
- Extensively Utilizing the Quite Room

any additional feedback not captured in the above questions.

Has your church created easily accessible pathways to navigate to the sanctuary and quite room?

- Yes
- No

Please select what summarizes the reason(s) why the church has not created easily accessible pathways to navigate to the sanctuary and quite room? If there are any other or additional reason(s) please specify in the space below.

- ☐ Perceived as **difficult** to implement for the church in creating a dementia-friendly space
- ☐ Perceived as **not being useful** to the church in creating a dementia-friendly space
- ☐ Perceived as **not being helpful** to the church in creating a dementia-friendly space
- ☐ [ ] Other or additional reason(s)

Since creating easily accessible pathways to navigate to your sanctuary and quite room, how helpful has this been for your faith-based organization (church and church members)?

- ○ Not at all Helpful
- ○ Slightly Helpful
- ○ Moderately Helpful
- ○ Very Helpful
- ○ Completely Helpful

When creating easily accessible pathways to navigate to your sanctuary and quite room, what was the level of difficulty for your faith-based organization (church and church members) to make and ensure accessible pathways?

- Extremely Difficult (not able to do so)
- Very Difficult
- Moderately Difficult
- Slightly Difficult
- Not Difficult at All (easy)

Since creating easily accessible pathways to navigate to your sanctuary and quite room, what best describes the level of uptake/usage of your faith-based organization (church and church members)?

- Not Utilizing the Pathways
- Somewhat Utilizing the Pathways
- Moderately Utilizing the Pathways
- Utilizing the Pathways
- Extensively Utilizing the Pathways

any additional feedback not captured in the above questions.

Has the church hosted and registered ministry leaders and senior pastors to attend the “Dementia-Friendly Workshop for Church Leaders” or similar workshop?

- ○ Yes
- ○ No

Please select what summarizes the reason(s) why the church has not hosted and registered ministry leaders and senior pastors to attend the “Dementia-Friendly Workshop for Church Leaders” or similar workshop? If there are any other or additional reason(s) please specify in the space below.

- ☐ Perceived as **difficult** to implement for the church in creating a dementia-friendly space
- ☐ Perceived as **not being useful** to the church in creating a dementia-friendly space

☐ Perceived as **not being helpful** to the church in creating a dementia-friendly space

☐ [ ] Other or additional reason(s)

Since hosting and registering ministry leaders and senior pastors to attend the "Dementia-Friendly Workshop for Church Leaders" or similar workshop, how helpful has this been for your faith-based organization (church and church members)?

○ Not at all Helpful

○ Slightly Helpful

○ Moderately Helpful

○ Very Helpful

○ Completely Helpful

When hosting and registering ministry leaders and senior pastors to attend the "Dementia-Friendly Workshop for Church Leaders" or similar workshop, what was the level of difficulty for your faith-based organization (church and church members) to make and ensure hosting and registration?

○ Extremely Difficult (not able to do so)

- Very Difficult
- Moderately Difficult
- Slightly Difficult
- Not Difficult at All (easy)

Since hosting and registering ministry leaders and senior pastors to attend the “Dementia-Friendly Workshop for Church Leaders” or similar workshop, what best describes the level of usage from what was learned at the workshops for your faith-based organization (church and church members)?

- Not Utilizing Anything Learned
- Somewhat Utilizing Workshop Activities and Lessons Learned
- Moderately Utilizing Workshop Activities and Lessons Learned
- Utilizing Workshop Activities and Lessons Learned
- Extensively Utilizing the Workshop Activities and Lessons Learned

any additional feedback not captured in the above questions.

Can you please give a brief summary of your church's experience with the *Required Partnership Activities and Modifications*? Any additional comments about the Required Modifications and Elements?

Offer Support Resources for Care Partners and Persons Living with Dementia

Thank you for completing the first portion of the program evaluation!

Take a 2-3 minute break! Move around a little before continuing the survey!

The following questions are aimed at evaluating the programs operations concerning "Offering support resources for care partners and persons living with dementia", that your church selected upon joining the ALTER program. (Part 2 out of 5)

Which of the following options did your church select (should be the same 3 or more options your church selected during enrollment of program):

- ☐ Organize a Memory Café
- ☐ Develop a dementia specific support group
- ☐ Build a resource library online or within the church
- ☐ Develop a respite program (Ex: offer at least 2 hours respite, twice per month)
- ☐ Offer a caregiver/care partner education program (Ex. self-care, The Great Village)
- ☐ Implement a social service coordination program
- ☐ ________ Other customization. Please specify

Offer Support Resources for Care Partners and Persons Living with Dementia

Answer the next set of related questions for the option your church implemented.

How long has the " ${lm://Field/1} " been implemented?

- Not yet implemented
- Less than a few weeks
- A few weeks
- 1-2 months
- 3-4 months
- 6 months- 1 year
- 1 year or more

Based on your churches selected resource " ${lm://Field/1} ", what was the level of difficulty for your faith-based organization (church and church members) to make, implement, and/or ensure your selected resource?

- Extremely Difficult (not able to do so)
- Very Difficult
- Moderately Difficult

- Slightly Difficult
- Not Difficult at All (easy)

Based on your churches selected resource " ${lm://Field/1} ", what best describes the level of uptake/usage of your faith-based organization (church and church members)?

- Not Utilizing
- Utilizing Twice a Year
- Utilizing Every Other Month
- Utilizing Once a Month
- Utilizing Weekly

Based on your churches selected resource " ${lm://Field/1} ", how helpful has this been for your faith-based organization (church and church members)?

- Not at all Helpful
- Slightly Helpful
- Moderately Helpful
- Very Helpful
- Completely Helpful

any additional feedback not captured in the above questions.

Offer Support Resources for Care Partners and Persons Living with Dementia

For the selected resource not chosen to implement at your church select what summarizes the reason(s) it was not chosen? If there are any other or additional reason(s) please specify in the space below.

" ${lm://Field/1} "

- ☐ Perceived as **difficult** to implement for the church in creating a dementia-friendly space
- ☐ Perceived as **not being useful** to the church in creating a dementia-friendly space
- ☐ Perceived as **not being helpful** to the church in creating a dementia-friendly space

☐ Other or additional reason(s)

Offer Support Resources for Care Partners and Persons Living with Dementia

Can you please give a brief summary of your church's experience with the *Support Resources for Care Partners and Persons Living with Dementia*? Any additional comments about the *Support Resources for Care Partners and Persons Living with Dementia*?

Provide or promote church and community education and awareness opportunities

Thank you for completing the second portion of the program evaluation!

Take a 2-3 minute break! Move around a little before continuing the survey!

The following questions are aimed at evaluating the programs operations concerning "Providing or promoting church and community education and awareness opportunities", that your church selected upon joining the ALTER program. (Part 3 out of 5)

Please select all the following options your church implemented (should be the same 2 or more options your church selected during enrollment of program):

- ☐ Adopt *Memory Sunday* initiative to spread awareness
- ☐ Host bi-annual dementia related education events (to include youth)
- ☐ Include updated dementia-related education and resources on website, mailings, church bulletin, and/or social media platforms
- ☐ Offer a simulated or virtual dementia experience session annually

Provide or promote church and community education and awareness opportunities

Answer the next set of related questions for the option your church implemented.

How long has the " ${lm://Field/1} " been implemented?

- Not yet implemented
- Less than a few weeks
- A few weeks
- 1-2 months
- 3-4 months
- 6 months- 1 year
- 1 year or more

Based on your churches selected resource " ${lm://Field/1} ", what was the level of difficulty for your faith-based organization (church and church members) to make, implement, and/or ensure your selected resource?

- Extremely Difficult (not able to do so)
- Very Difficult
- Moderately Difficult
- Slightly Difficult
- Not Difficult at All (easy)

Based on your churches selected resource " ${lm://Field/1} ", what best describes the level of uptake/usage of your faith-based organization (church and church members)?

- ○ Not Utilizing
- ○ Utilizing Twice a Year
- ○ Utilizing Every Other Month
- ○ Utilizing Once a Month
- ○ Utilizing Weekly (or as specified in the selected resource)

Based on your churches selected resource " ${lm://Field/1} ", how helpful has this been for your faith-based organization (church and church members)?

- ○ Not at all Helpful
- ○ Slightly Helpful
- ○ Moderately Helpful
- ○ Very Helpful
- ○ Completely Helpful

any additional feedback not captured in the above questions.

Provide or promote church and community education and awareness opportunities

For the selected resource not chosen to implement at your church select what summarizes the reason(s) it was not chosen? If there are any other or additional reason(s) please specify in the space below.

" ${lm://Field/1} "

- ☐ Perceived as **difficult** to implement for the church in creating a dementia-friendly space
- ☐ Perceived as **not being useful** to the church in creating a dementia-friendly space
- ☐ Perceived as **not being helpful** to the church in creating a dementia-friendly space
- ☐ Other or additional reason(s)

Provide or promote church and community education and awareness opportunities

Can you please give a brief summary of your church's experience with *Providing or Promoting Church and Community Education and Awareness Opportunities*? Any additional comments about *Providing or Promoting Church and Community Education and Awareness Opportunities*?

Offer an inclusive and/or modified worship experience at least once a month

Thank you for completing the third portion of the program evaluation!

Take a 2-3 minute break! Move around a little before continuing the survey!

The following questions are aimed at evaluating the programs operations concerning "Offering an inclusive

and/or modified worship experience at least once a month", that your church selected upon joining the ALTER program. (Part 4 out of 5)

Please select all the following options your church implemented (should be the same 3 or more options your church selected during enrollment of program):

- ☐ Shorten service length: 75 minutes or less
- ☐ Simplify sermon related to familiar biblical stories using visual aids
- ☐ Include short, familiar prayers and Bible verses (Ex: The Lord's Prayer)
- ☐ Memory support staff present (Ex: ushers, hospitality, health/wellness that have received training)
- ☐ Offer a higher proportion of music (familiar hymns and songs are highly recommended)
- ☐ Modify order of worship service: Praise and Worship, Prayer, Sermon, and Announcements
- ☐ [ ] Other customization

Offer an inclusive and/or modified worship experience at least once a month

Answer the next set of related questions for the option your church implemented.

How long has the " ${lm://Field/1} " been implemented?

- Not yet implemented
- Less than a few weeks
- A few weeks
- 1-2 months
- 3-4 months
- 6 months- 1 year
- 1 year or more

Based on your churches selected resource " ${lm://Field/1} ", what was the level of difficulty for your faith-based organization (church and church members) to make, implement, and/or ensure your selected resource?

- Extremely Difficult (not able to do so)
- Very Difficult
- Moderately Difficult
- Slightly Difficult
- Not Difficult at All (easy)

Based on your churches selected resource " ${lm://Field/1} ", what best describes the level of usage of your faith-based organization (church and church members)?

- Not Utilizing
- Utilizing Twice a Year
- Utilizing Every Other Month
- Utilizing Once a Month
- Utilizing Weekly

Based on your churches selected resource " ${lm://Field/1} ", how helpful has this been for your faith-based organization (church and church members)?

- Not at all Helpful
- Slightly Helpful
- Moderately Helpful
- Very Helpful
- Completely Helpful

any additional feedback not captured in the above questions.

Offer an inclusive and/or modified worship experience at least once a month

For the selected resource not chosen to implement at your church select what summarizes the reason(s) it was not chosen? If there are any other or additional reason(s) please specify in the space below.

" ${lm://Field/1} "

- ☐ Perceived as **difficult** to implement for the church in creating a dementia-friendly space
- ☐ Perceived as **not being useful** to the church in creating a dementia-friendly space
- ☐ Perceived as **not being helpful** to the church in creating a dementia-friendly space
- ☐ Other or additional reason(s)

Offer an inclusive and/or modified worship experience at least once a month

Can you please give a brief summary of your church's experience with *Offering an Inclusive and/or Modified Worship Experience at Least Once a Month*? Any additional comments about *Offering an Inclusive and/or Modified Worship Experience at Least Once a Month*?

Other customized activities

Thank you for completing the fourth portion of the program evaluation!

Take a 2-3 minute break! Move around a little before continuing the survey! (OPTIONAL BREAK)

The following questions are aimed at evaluating the programs operations concerning the "Other customized activities", that your church selected upon joining ALTER. (Part 5 out of 5)

Below are optional questions to answer. If your church specified any other customized activities please move on the the next portion of the survey by selecting "Yes." If you did not specify any additional activities then please skip the last section and submit the survey by selecting "No!"

Has your church implemented any other customized activities?

- Yes
- No

Please write in one of the "Other customized activities", that your church came up with upon joining the Dementia-Friendly Faith Village Community Program.

Please write in one of the "Other customized activities", that your church came up with upon joining the Dementia-Friendly Faith Village Community Program.

Finished 1

Thank you so much for completing the survey and helping our team understand how the ALTER program can be improved!!

Other customized activities

How long has " ${q://QID75/ChoiceTextEntryValue} " been implemented?

- Not yet implemented
- Less than a few weeks
- A few weeks
- 1-2 months
- 3-4 months
- 6 months- 1 year
- 1 year or more

Based on your churches selected resource " ${q://QID75/ChoiceTextEntryValue} ", what was the level of difficulty for your faith-based organization (church and church members) to make, implement, and/or ensure your selected resource?

- Extremely Difficult (not able to do so)
- Very Difficult
- Moderately Difficult
- Slightly Difficult
- Not Difficult at All (easy)

Based on your churches selected resource " ${q://QID75/ChoiceTextEntryValue} ", what best describes the level of uptake/usage of your faith-based organization (church and church members)?

- Not Utilizing
- Utilizing Twice a Year
- Utilizing Every Other Month
- Utilizing once a month
- Utilizing weekly

Based on your churches selected resource " ${q://QID75/ChoiceTextEntryValue} ", how helpful has this been for your faith-based organization (church and church members)?

- Not at all Helpful
- Slightly Helpful
- Moderately Helpful
- Very Helpful
- Completely Helpful

Explain why your church chose to create a different activity or modification to implement?

How long has " ${q://QID76/ChoiceTextEntryValue} " been implemented?

- Not Applicable
- Not yet implemented
- Less than a few weeks
- A few weeks
- 1-2 months
- 3-4 months
- 6 months- 1 year
- 1 year or more

Based on your churches selected resource " ${q://QID76/ChoiceTextEntryValue} ", what was the level of difficulty for your faith-based organization (church and church members) to make, implement, and/or ensure your selected resource?

- Extremely Difficult (not able to do so)
- Very Difficult
- Moderately Difficult
- Slightly Difficult
- Not Difficult at All (easy)

Based on your churches selected resource " ${q://QID76/ChoiceTextEntryValue} ", what best describes the level of uptake/usage of your faith-based organization (church and church members)?

- Not Utilizing
- Utilizing Twice a Year
- Utilizing Every Other Month
- Utilizing Once a Month
- Utilizing Weekly

Based on your churches selected resource " ${q://QID76/ChoiceTextEntryValue} ", how helpful has this been for your faith-based organization (church and church members)?

- Not at all Helpful
- Slightly Helpful
- Moderately Helpful
- Very Helpful
- Completely Helpful

Explain why your church chose to create a different activity or modification to implement?

Finished

Thank you so much for completing the survey and helping our team understand how the ALTER program can be improved!!

ALTER

Powered by Qualtrics
